# Organoids From Mucinous Appendiceal Adenocarcinomas as High-Fidelity Models for Individual Therapy

**DOI:** 10.3389/fmed.2022.829033

**Published:** 2022-06-02

**Authors:** Guangyao Liu, Xing Xiao, Yujian Xia, Weibing Huang, Wei Chen, Jiannan Xu, Songyao Chen, Huijin Wang, Jitao Wei, Huan Li, Man Shu, Xiaofang Lu, Changhua Zhang, Yulong He

**Affiliations:** ^1^Digestive Diseases Center, The Seventh Affiliated Hospital of Sun Yat-sen University, Shenzhen, China; ^2^Department of Gastrointestinal Surgery, Northern Jiangsu People's Hospital, Yangzhou, China; ^3^Department of Pathology, The First Affiliated Hospital, Sun Yat-sen University, Guangzhou, China; ^4^Department of Pathology, The Seventh Affiliated Hospital of Sun Yat-sen University, Shenzhen, China; ^5^Department of Gastrointestinal Surgery, The First Affiliated Hospital, Sun Yat-sen University, Guangzhou, China

**Keywords:** mucinous appendiceal adenocarcinoma, organoid culture, individualized therapy, drug sensitivity test, chemotherapy

## Abstract

**Background:**

Mucinous appendiceal adenocarcinoma (MAA) is a rare, heterogeneous disease. Patients with unrespectable mucinous appendiceal adenocarcinoma presenting with peritoneal spread are treated by intraperitoneal chemotherapy, hyperthermic intraperitoneal chemotherapy, systemic chemotherapy, or targeted therapy. However, there are no guidelines for efficacious drugs against mucinous appendiceal adenocarcinoma. Therefore, relevant high-fidelity models should be investigated to identify effective drugs for individual therapy.

**Methods:**

Surgical tumor specimens were obtained from a mucinous appendiceal adenocarcinoma patient. The tissue was digested and organoid culture was established. H&E and immunohistochemistry staining as well as DNA sequencing was performed on tissue and organoid. The pathological characteristics and gene mutations of the organoid were compared to those of the original tumor. Drug sensitivity tests were performed on organoid and the patient clinical responds to chemotherapy and targeted therapy was compared.

**Results:**

Organoids were successfully established and stably passaged. Pathological characteristics of organoids including H&E staining and expression of protein markers (CK20, CDX-2, STAB2, CD7, PAX8) were consistent to those of the original tumor. Moreover, the organoids carried the same gene mutations as the primary tumor. Sensitivity of the organoids to chemotherapeutic drugs and tyrosine kinase inhibitors included: 5-FU (IC_50_ 43.95 μM), Oxaliplatin (IC_50_ 23.49 μM), SN38 (IC_50_ 1.02 μM), Apatinib (IC_50_ 0.10 μM), Dasatinib (IC_50_ 2.27 μM), Docetaxel (IC_50_ 5.26 μM), Regorafenib (IC_50_ 18.90 μM), and Everolimus (IC_50_ 9.20 μM). The sensitivities of organoid to these drugs were comparable to those of the patient's clinical responses.

**Conclusion:**

The mucinous appendiceal adenocarcinoma organoid model which retained the characteristics of the primary tumor was successfully established. Combined organoid-based drug screening and high throughput sequencing provided a promising way for mucinous appendiceal adenocarcinoma treatment.

## Introduction

Primary cancers of the appendix are rare, with an age-adjusted incidence of 0.12 cases per 1 million individuals per year (1). Primary appendiceal cancers are histologically diverse and the classification is complex and controversial. According to tissue of origin and morphological features, primary appendiceal cancers are subcategorized as colonic-type adenocarcinoma, mucinous adenocarcinoma, goblet cell adenocarcinoma, and neuroendocrine carcinoma (2). Mucinous appendiceal adenocarcinoma (MAA) arises from an adenomatous polyp or serrated adenoma (3). Over 50% of MAA patients present with pseudomyxoma peritonei at diagnosis, indicating worse prognostic outcomes (4). The treatment for MAA includes resection of the primary site, intraperitoneal chemotherapy, hyperthermic intraperitoneal chemotherapy (HIPEC), systemic chemotherapy (SC) or targeted therapy (5). Systemic chemotherapy and targeted therapy are the only therapeutic option for stage IV patients with distant metastases (6). The current systemic therapy for MAA is based on the guideline for colorectal cancer (CRC) suggesting revisions are required in National Comprehensive Cancer Network (NCCN) guideline (7). Meanwhile, some retrospective studies also suggest that appropriate regimes for MAA treatment need to be further studied (6, 8, 9).

The genetic background of MAA and colorectal cancer are very different. CRC and MAA have been extensively studied by Next-generation sequencing (NGS) (10, 11). Compared to colorectal cancer, *APC* gene mutations are common in CRC but rare in MAA. Concurrent activation of the *KRAS* and *GNAS* mediated signaling pathways in MAA is more similar to pancreatic intraductal papillary mucinous neoplasm than CRC (10). Therefore, identification of optimal drugs for MAA treatment has been a challenge.

Patient-derived cell lines or xenografts have been widely used to predict the effectiveness of chemotherapies. However, long turn-around times, poor scalability, and low success rates limit their clinical applications (12, 13). Patient-derived tumor organoids (PDOs) are 3D culture of tumor cells that are derived from individual patients. Due to the high success rates, indefinite expansion, as well as morphological and genetic features that resemble those of the original tumor, PDOs have been extensively used in colorectal cancer studies (14). Recently a study reported MAA tumor cells can be cultured into organoids (15). However, more research is still needed. Firstly, consistency between primary tumors and organoids should be explored further. Secondly, as a preclinical model, combined MAA organoid-based drug screening and high throughput sequencing could be a feasible way for personalized therapy. In addition, a parallel analysis of drug sensitivities between MAA and clinical response of the corresponding patient is needed to demonstrate whether the MAA organoid could guide individual therapy. In this study, we describe a case of a 53-year-old Chinese woman with MAA, whose tumor organoids were established and used to evaluate drug sensitivity. To the best of our knowledge, this is the first extensive study of an MAA organoid which retained the morphology and genetic aberrations of the original tumors. In addition, this study combined MAA organoid-based drug screening and high throughput sequencing, which provides a promising way for MAA treatment.

## Materials and Methods

### Specimen Collection and Clinical Data

This study was approved by the ethics committee of the Seventh Affiliated Hospital of Sun Yat-sen University. An informed consent from the patient was obtained before tissue collection. Patient information and associated clinical information was de-identified (Ethics approval document number: KY-2020-042-01). Tumor tissues were obtained from the abdominal wall stoma metastasis of a patient, who had been diagnosed with MAA. A normal tissue (5 cm away from the tumor margin) was also obtained. Some excised tissue was fixed with formalin for pathological analysis, while some tissue was snap frozen with liquid nitrogen and stored at −80°C for DNA sequencing. Clinical data of the patient were evaluated by an oncologist.

### Establishment of the Organoid

The excised tissue was placed in antibiotic-containing Dulbecco's MEM (Gibco, CAT#61965-026) and transferred at 4°C within half an hour. The tissue was washed three times using antibiotic-supplemented-PBS. The tissue was cut into small pieces and digested with 5 mg/ml type IV collagenase (MP, CAT#219511080) and 0.25% Trypsin-EDTA (Gibco, CAT#25200-072) for 30 min. Whole blood red cell lysing reagent (Solarbio, CAT#R1010) was used to remove red blood cells. The resulting suspension was incubated at 37°C for 1 h, after which the isolated suspension was resuspended with DMEM supplemented with 10% fetal bovine serum (Gibco, CAT#C11995500BT) and centrifuged at 300 × *g* for 5 min. The obtained cells were mixed with the matrigel (Corning, CAT#356231) on ice at a ratio of 5 × 10^5^ cells per 100 μl. Then, the mixture was seeded on a 96-well plate. After incubation at 37°C for 15 min, 500 μl Intesticult ™ Organoid Growth Medium (STEM CELL, CAT#06010) and 0.5 μl 10mM Y27632 (Tagertmol, CAT#T1870) were added to each well. Organoid cultures were maintained in a 5% CO_2_ atmosphere at 37°C. The medium was changed every 2 days and subcultured once a week.

For subculture, the organoid was passaged at a split ratio of 1:2 to 1:3 according the number of organoids. The culture medium was removed and organoid was washed with PBS. One ml of Tryple Express (Gibco, CAT#12604013) was added into the well and incubated for 30 min in a 5% CO_2_ atmosphere at 37°C. After more than 80 percent of the organoids were digested into single cells, DMEM with 10%FBS was added to neutralize the enzymes. Cells were centrifuged at 300 × g for 5 min. Then the precipitation cells were resuspended in cold DMEM and mixed with Matrigel, and then reseeded as described above.

### Immunohistochemistry

Original tumor tissues were fixed in 10% Neutral buffer formalin and embedded in paraffin blocks using standard procedures. For immunohistochemistry of the organoids, cell recovery solution (Corning, CAT#354270) was added to the medium to dissolve the Matrigel for 1 h, centrifuged at 300 × g for 5 min at room temperature, fixed at 37°C in 10% formalin for 2 h, and embedded in paraffin. Then, 5 μm micron sections were used for hematoxylin and eosin (H&E) staining. Primary antibodies used in this study include: CDX2 (DAKO, CAT#M3636, 1/50), CK20 (DAKO, CAT#M7019, 1/100), PAX8(ORIGENE, CAT#TA327724, 1/100), STAB2(ORIGENE, CAT#CF806750, 1/150), KI67(Affinity Biosciences, CAT#AF5208, 1/100), CD44(Proteintech, CAT#15675, 1/200), CK7 (DAKO, CAT#M7018, 1/200), and PDL1 (DAKO, CAT#M3653, 1/100). The DAB system (Biyuntian Biotechnology, CAT#A0288) was used for immunohistochemistry. Imaging was performed by a Leica DMI8 microscope at a magnification of ×100 (×10 objective and ×10 eyepiece) and ×400(×40 objective and ×10 eyepiece). Images were scanned by K-Viewer (Jiang Feng company, China). Two pathologists, who were blinded, scored each tissue.

### DNA Sequencing and Bioinformatics Analysis

Genomic DNA from normal and tumor tissues as well as organoids were extracted using Magnetic Universal Genomic DNA Kit (TIAN GEN, CAT#DP705). Sample integrity and purity were detected by agarose gel electrophoresis (concentration of agarose gel: 1%; voltage: 150 V and electrophoresis time: 40 min). Exome sequences were enriched using an Agilent liquid capture system based on the manufacturer's guidelines (Agilent Sure Select Human All Exon V6). DNA libraries were sequenced on a BGISEQ-500 platform. After sequencing, quality control, mapping and counting were done using R-project and maftools (16).

### Drug Screening

The organoid was dissected into single cells, which were re-suspended in Matrigel on ice and seeded into 96-well-plates with about 1×10^4^ cells/well. Then, 100 μl Intesticult ™ Organoid Growth Medium (STEM CELL, CAT#06010) was added to each well. 2 days later, culture medium was replated containing different drugs (5-FU, oxaliplatin, SN38, apatinib, dasatinib, docetaxel, regorafenib, and everolimus) and dimethyl sulfoxide (DMSO) was used as the negative control. Each drug was tested in 6 serial diluted concentrations and three replicates. Images of living cultures were obtained daily. After 3 days, Cell counting kit-8 (Dojindo, CAT#CK04) was used to measure the cell viability following the manufacturer's instruction. IC_50_ values were calculated using GraphPad Prism 8. Drug tests were performed three times.

## Results

### Case Report

**Figure 1:**
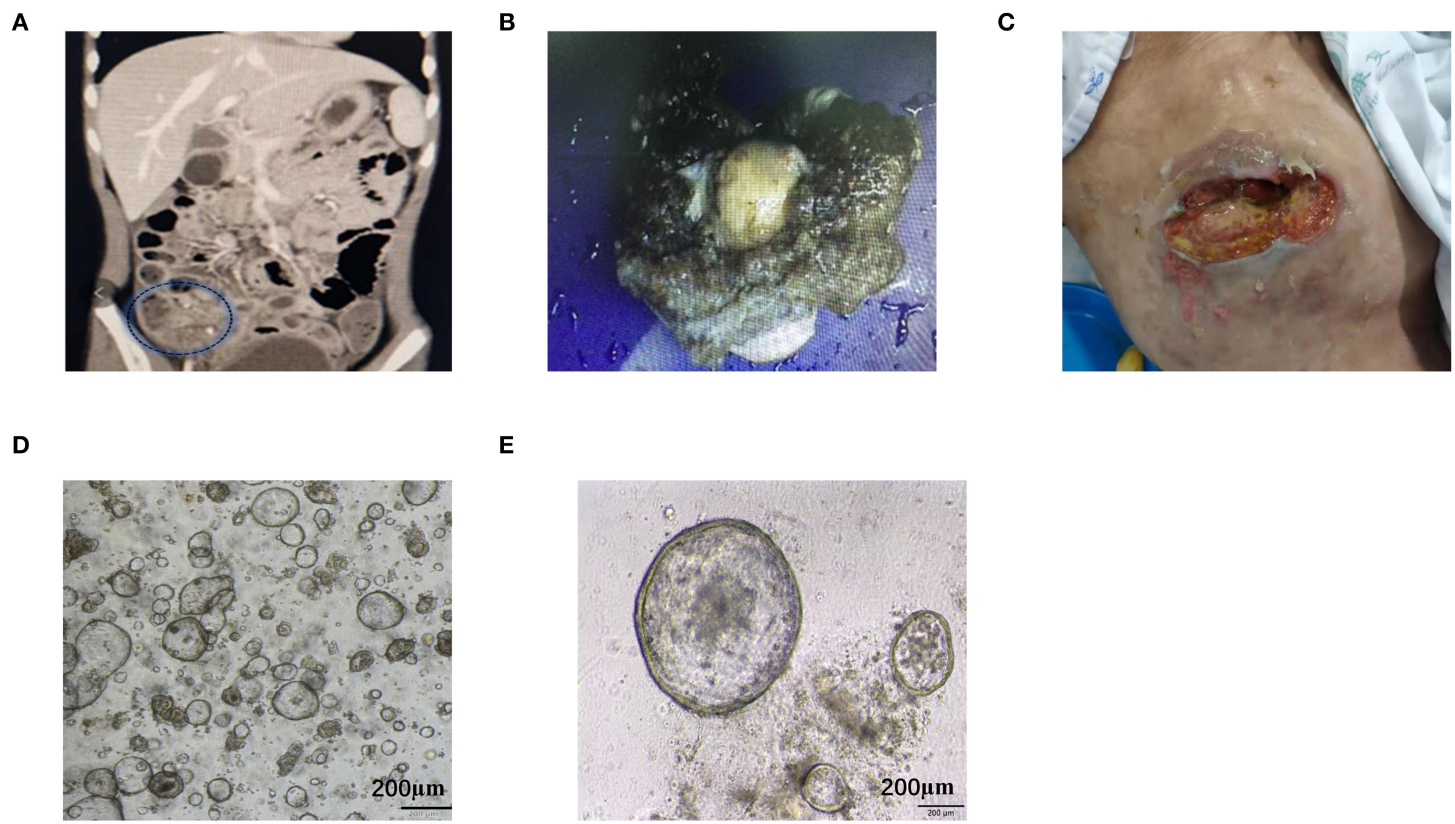
Representative images of the clinical course of the MAA patient. **(A)** Abdominal CT scan showed right lower abdominal mass with ascites. **(B)** Intraoperative specimens suggested that the tumor had invaded the serous membrane with massive mucinous infiltrations. **(C)** Metastatic tumor in abdominal wall stoma. **(D,E)** Stably passaged organoids appear spherical with different sizes. Scale bar = 200 μm.

The patient was a 53-year-old Chinese woman. She was admitted to the hospital with right lower abdominal pain. CT examination showed a tumor mass in the right lower abdomen (Figure 1A). Exploratory laparotomy confirmed the right lower abdomen tumor, which envelopes the appendix and right ovary, with a large amount of mucinous infiltration in the abdominal cavity (Figure 1B). Right hemicolectomy, oophorectomy, and ostomy were performed on the patient. During surgery, oxaliplatin HIPEC was administered. Post-operative pathology result reported moderately differentiated mucinous adenocarcinoma of the appendix, and mesenteric lymph node LN 0/14, the abdominal mucus contains tumor cells (AJCC TNM stage: PT4bN0M1b, IVb). After 2 years, a metastatic tumor was found in the abdominal wall stoma (Figure 1C). CT examination revealed heart diaphragm angle lymph node metastasis. Systemic chemotherapy was prescribed with seven courses of oxaliplatin and 5-FU (XELOX regime). After 5 months, CT scan revealed that the sum of the length and width of heart diaphragm angle lymph node had increased, evaluated progressive disease (PD) of the tumor according to RECIST (Response Evaluation Criteria in Solid Tumors) 1.1 criteria (17). The patient was prescribed with a new regime of chemotherapy. Four courses of irinotecan and 5-FU (FORFIRI regime) were administered, which led to a decrease in the lymph node, indicating stable disease (SD) of the tumor. To improve treatment outcomes, three courses of apatinib were administered. CT scans found that the lymph node had significantly reduced, implying PR (partial response).

### Establishment of the MAA Organoid

A tumor biopsy was performed on the abdominal wall metastasis of patient before chemotherapy. Biopsy was performed near the tumor center to avoid normal tissue contamination. The protocol for MAA organoid culture was similar to colon cancer organoid as described by Hans Clevers (11). Tumor tissue was digested and the isolated tumor cells were mixed with Matrigel and seeded onto a 96-well-plate. Cells were cultured in Intesticult™ Organoid Growth Medium, which could also support for long-term culture. MAA organoid was passaged once every week. The organoid exhibited loose spherical structures, with a doubling time of ~72 h. The organoid line was grown for over 14 months, with high proliferation rate and stable passaging. Stable passaging organoid was cryopreserved and stored in the biobank for future using (Figures 1D,E; Supplementary Figure S1A).

### Pathological Characteristics of the Organoid and Tumor

**Figure 2:**
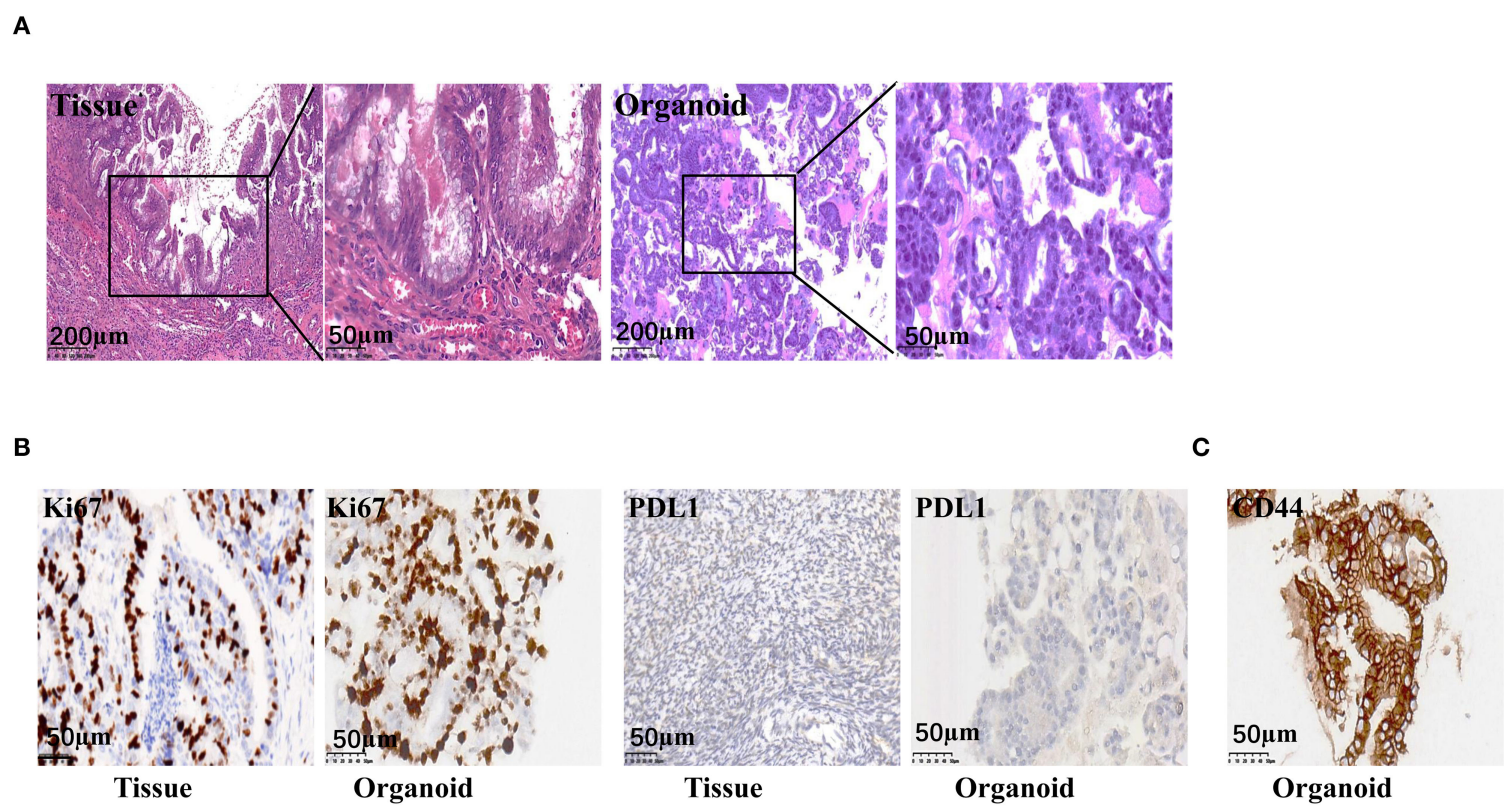
Representative images of the pathological staining of MAA. **(A)** H&E staining of the organoid (passage 8) and the original tumor. **(B)** Immunohistochemistry staining of the original tumor and the organoid (passage 8) for Ki67 and PDL-1. **(C)** Immunohistochemistry staining of the organoid (passage 8) for CD44.

Tumor pathology analyses was performed after tumor removal. Pathological analyses of organoids were conducted at passage eight. The H&E staining of tumor tissue showed the epithelium consists of tall columnar mucinous cells with a small amount of mucus and enlarged hyperchromatic nuclei. The nuclear membrane was irregular and the karyokinesis was increased. The organoid H&E result showed high cell density with varied cell sizes. The nuclei were large with deep dyeing, and the nucleoplasmic ratio was higher. The organoid retained pathological characteristics of the tumor tissue (Figure 2A). The organoid showed negative programmed cell death-Ligand-1 (PDL1) and strong positive Ki67 expression, which was consistent with that observed in the tumor (Figure 2B). The MAA organoid showed strong positive staining of CD44, indicating that it was stem cell derived (Figure 2C).

**Figure 3:**
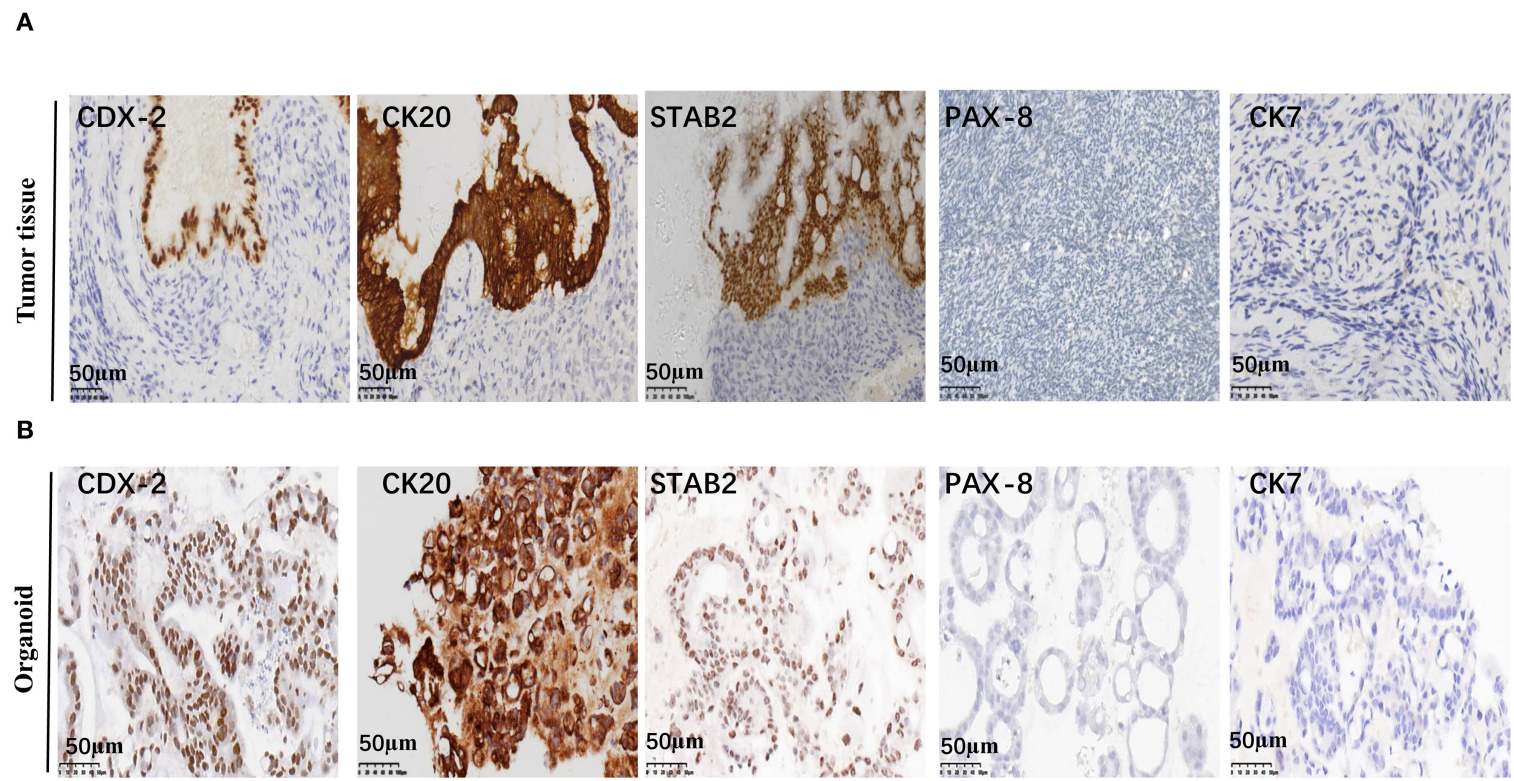
Representative immunohistochemistry staining of MAA. Representative images of CDX2, CK20, STAB2, PAX-8, and CK7 immunohistochemical staining of the original tumor **(A)** and organoid (passage 8) **(B)** Scale bar = 50 μm.

Mucinous appendiceal adenocarcinoma and ovarian mucinous tumors are very similar morphologically. It may be difficult to distinguish ovarian involvement by a MAA and a primary ovarian mucinous tumor, particularly when the latter is associated with mucinous ascites. They are treated differently since a primary carcinoma confined to the ovary will not usually warrant chemotherapy, whereas patients with MAA involving the ovary have a poor prognosis and may need systemic treatment (18). Differentiating between these two tumor types is a challenge for pathologists (19). Thus, a series of immunohistochemical staining was performed to confirm the tumor origin. Positive staining of CK20, CDX-2, STAB2, and negative staining of CK7 and PAX-8 was observed for the tumor tissue, indicating that the tumor was of gastrointestinal adenocarcinoma origin, rather than ovarian origin. Consistently, the organoid also showed positive staining of CK20, CDX-2, STAB2, and negative staining of CK7 and PAX-8 (Figures 3A,B; Supplementary Figure S1B).

### Gene Sequencing

**Figure 4:**
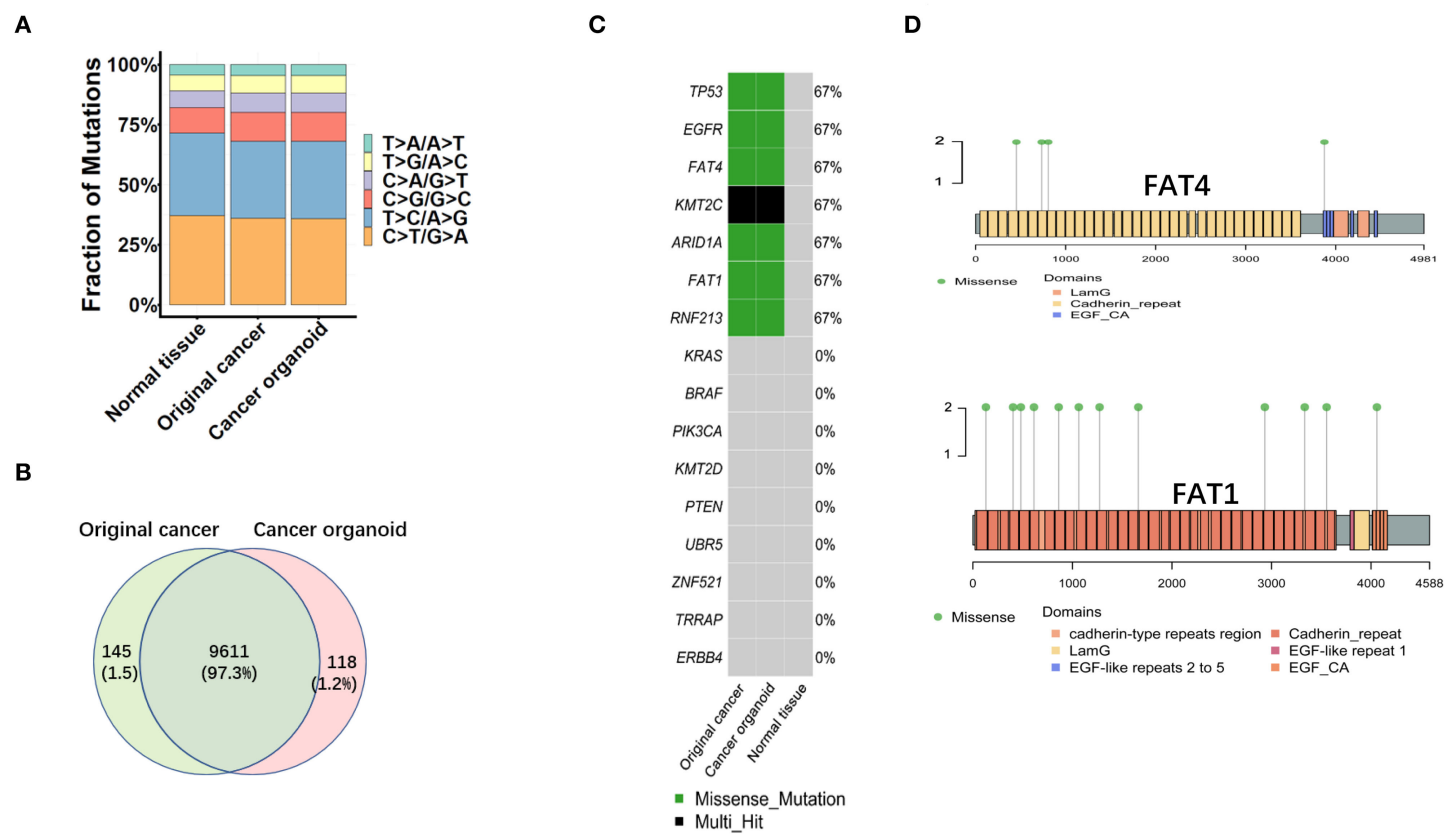
Genetic characterization of MAA. **(A)** Distribution of base substitutions in the normal, original, and organoid tissue. **(B)** Venn diagram showing a 97.3% overlap of single nucleotide variants between the original tissue and cancer organoid. **(C)** Representative gene mutations in the organoid and original tissue. **(D)** Functionally altered regions of FAT4 and FAT1 genes encoding amino acids of the original tumor and the organoid.

To detect gene mutations of the tumor and to determine whether the organoid had the same gene mutations as the primary tumor, whole exome sequencing (WES) was performed. Circos plots of the original tissues and cancer organoids exhibited a similar distribution pattern (Supplementary Figure S2). Base substitution distribution in the original tissue and organoid is shown in Figure 4A. Compared to normal tissue, both tissues and organoid showed fewer base substitutions of T>C/A>G and C>T/G>A, and more base substitutions of C>G/G>C and C>A/G>T (Figure 4A). A total of 9,611 gene mutations were found in the tumor organoid, accounting for 97.31% of the 9,756 gene mutations in the original tissue (Figure 4B). Mutations in MAA*TP53, EGFR, FAT4, KMT2C, ARID1A, FAT1*, and *RNF213* were observed in the original cancer tissues, whereas *KRAS* and *BRAF* mutations were not identified (Figure 4C). In addition, the organoids carried the same mutations as the original tumor. Further analysis revealed that the organoids matched the tumor in the number of mutated genes while functional region alterations in the original tumor amino acids were preserved (Figure 4D).

### Drug Sensitivity Tests Using the Organoid

**Figure 5:**
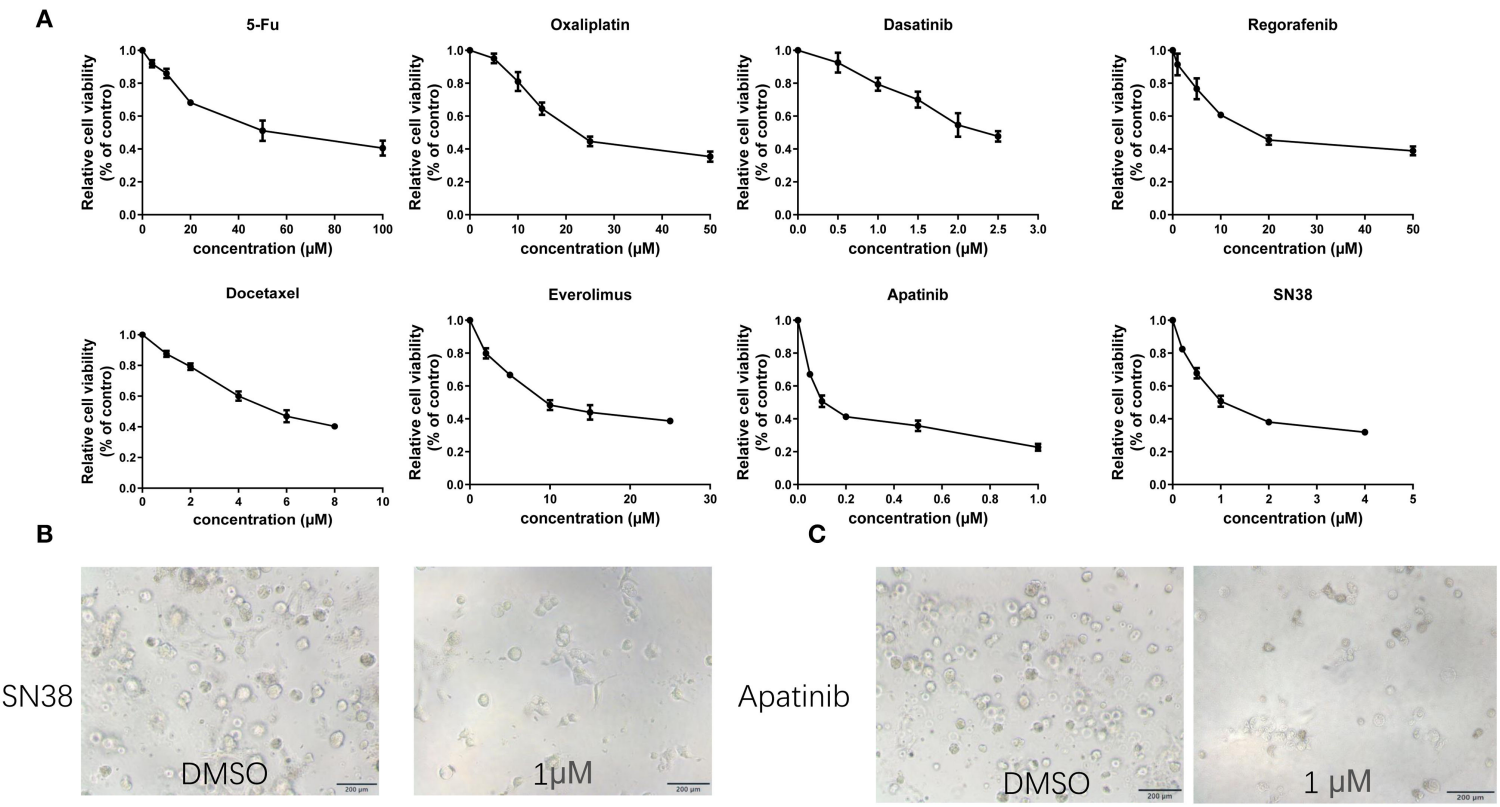
Representative images and drug tests of the organoids. **(A)** Dose-response curves of organoids treated with 5-FU, oxaliplatin, SN38, apatinib, dasatinib, docetaxel, regorafenib and everolimus. **(B)** Mean ± SD of results from three independent experiments is shown for each drug. **(C)** Representative images of the organoid after treatment with 1 μM SN38 and DMSO. Organoid treated with 1 μM apatinib and DMSO. Scale bar = 200 μm.

After 2 months, MAA organoids can be stable passaged and the passage 8–10, was used for drug screening. Based on the CRC guideline, traditional chemo drugs, including 5FU, oxaliplatin, and SN38 were used. In addition, small molecular targeted agents approved for treatment of gastrointestinal malignancies including apatinib, dasatinib, docetaxel, regorafenib, and everolimus were evaluated (Figure 5A). Sensitivities of the organoids to chemotherapeutic drugs and tyrosine kinase inhibitors were: 5-FU (IC_50_ 43.95 μM, 95%CI 34.52–55.97), oxaliplatin (IC_50_ 23.49 μM, 95%CI 20.85–26.46), SN38 (IC_50_ 1.02 μM, 95%CI 0.71–1.33), apatinib (IC_50_ 0.10 μM, 95%CI 0.06–0.20), dasatinib (IC_50_ 2.27 μM, 95%CI 2.04–2.50), docetaxel (IC_50_ 5.26 μM, 95%CI 3.63–6.89), regorafenib (IC_50_ 18.90 μM, 95%CI 10.84–26.97), and everolimus (IC_50_ 9.20 μM, 95%CI 3.63–14.79) (Table 1). Of all drugs tested, apatinib (IC_50_ 0.10μM, 95%CI 0.06–0.20) and SN38(1.02μM, 95%CI 0.71–1.33) showed the best anti-cancer effect (Figures 5B,C).

**Table 1:** IC_50_ values for each drug and confidence intervals (CIs) for the organoid.

**Drug**	**IC_50_ (μM)**	**95%CI(μM)**
5-FU	43.95	34.52–55.97
Oxaliplatin	23.49	20.85–26.46
Apatinib	0.10	0.06–0.20
SN38	1.02	0.71–1.33
Dasatinib	2.27	2.04–2.50
Docetaxel	5.26	3.63–6.89
Regorafenib	18.90	10.84–26.97
Everolimus	9.20	3.63–14.79

### Consistency Between Organoid and Patient Responses to Chemotherapeutic Drugs

**Figure 6:**
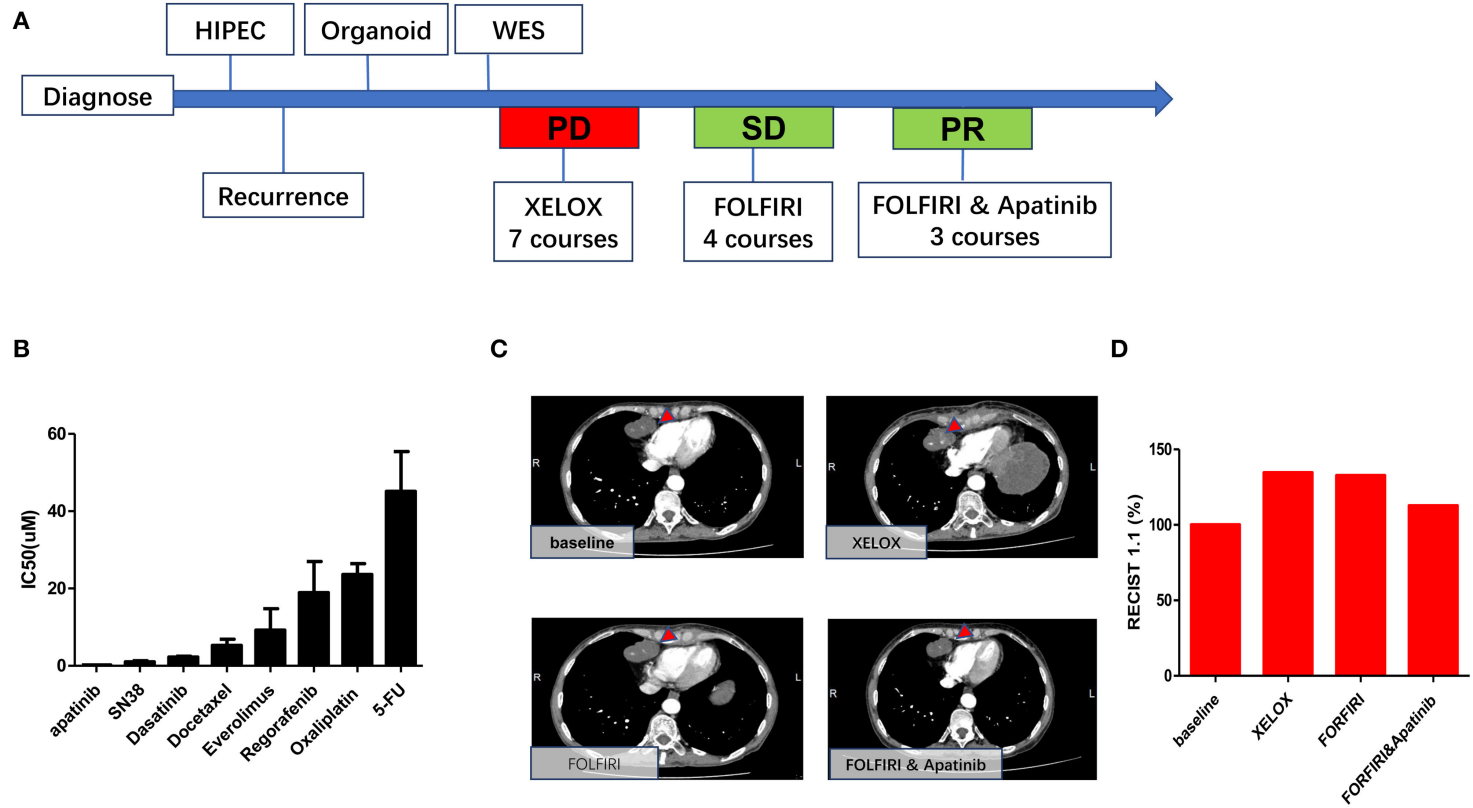
Consistency between organoid and patient responses to different drugs. **(A)** Timetable of disease progression. **(B)** Drug sensitivity data indicating low sensitivities to 5-fu and oxaliplatin and high sensitivities to apatinib and SN38. **(C)** Representative CT images of heart diaphragm angle lymph node after treatment. **(D)** Sum of the length and width of heart diaphragm angle lymph node after treatment with XELOX, FORFIRI, and FORFIRI and apatinib, red bars indicate volume of the target metastasis [according to RECIST 1.1].

As shown in Figures 6A,B, significantly higher IC_50_ values for 5FU (IC_50_ 43.95 μM, 95%CI 34.52–55.97) and oxaliplatin IC_50_ (IC_50_ 23.49 μM, 95%CI 20.85–26.46) were observed for the organoid, indicating drug resistance. Similarly, the patient was evaluated as PD, as indicated by enlargement of heart diaphragm angle lymph node after oxaliplatin and 5-FU chemotherapy (XELOX), according to RECIST 1.1 criteria (Figures 6C,D). On the other hand, SN38, the primary bioactive metabolite of irinotecan, had a lower IC_50_ (1.02 μM, 95%CI 0.71–1.33) in the MAA organoid. This is consistent with the tumor regression after FOLFORI chemotherapy (Figures 6C,D). Of all drugs tested, apatinib (IC_50_ 0.10μM, 95%CI 0.06–0.20), an inhibitor of angiogenesis, showed the lowest IC_50_ value. Consistently, after apatinib treatment, chest CT imaging showed that the tumor shrank further.

## Discussion

Mucinous appendiceal adenocarcinoma is a rare tumor that originates from the adenomatous polyp or serrated adenoma. It frequently presents with the pseudomyxoma peritonei syndrome. It is common in middle-aged and older women (3). Early tumors are usually found in acute appendicitis surgical samples and at advanced stages, often in combination with ascites, which mainly presents as abdominal pain and abdominal distension (2). The patient was a 53-year-old Chinese female presenting with a right lower abdominal pain. A massive mucinous tissue was found in the abdominal cavity of the patient. H&E staining showed middle differentiated mucinous adenocarcinoma, consistent with MAA characteristics. Primary ovarian mucinous tumor was top on the differential diagnosis for MAA. Patients with MAA involving the ovary have a poor prognosis and may need systemic treatment. Several protein markers including CK20, CDX-2, STAB2, CK7, PAX-8 were usually used to differentiate these two tumors (19). Our result showed that the tumor tissue was positive in CK20, CDX-2, STAB2, and negative in CK7 and PAX8, which confirmed the appendix origin.

A few, mostly single-institution studies, have evaluated the treatment outcomes of MAA and suggested individualized therapy for MAA (6, 8, 9). MAA gene sequencing revealed tumor heterogeneities, as reflected by high number and variations of mutations. This explains for the same kind of tumor response to different drugs (20, 21). Further studies on gene mutations in MAA focusing on individualized treatment may improve future treatment outcomes (2, 9). The selection of an effective preclinical model for MAA is necessary. However, a limited number of preclinical studies on MAA tumors have been done (22, 23). Due to convenience and reliable growth, tumor cell lines have been widely used in preclinical research; however, 2D culture models do not mimic tumor microenvironments and homogenization significantly affects chemotherapeutic outcomes (12). In addition, MAA derived primary cells are not suitable for high-throughput experiments because of their slow growth rate and poor viability (22). Although PDX has been shown to have the potential for evaluating drug resistance mechanisms and identifying new therapies, it is limited by high costs and long culture cycles (13). PDOs have shown a bright future in the treatment of gastrointestinal cancer (24). Recently, research reported establishment of MAA organoids (15). However, this study did not report the long-term passaging ability of their models. In addition, the consistency between primary tumors and organoids was not explored in this study. In our study, the MAA organoid was successfully established and stably cultured for several months. We demonstrated the consistency between primary tumor and organoids in terms of pathological characteristics and gene mutations. On the other hand, as a preclinical model, it is necessary to test whether the drug sensitivity of organoid is consistent with clinical treatment. In this study, drug sensitivity analysis showed high IC_50_ values for 5-FU and oxaliplatin, consistent with progression of patient after XELOX treatment. This also implied traditional chemotherapy for colorectal cancer may not be appropriate for MAA (8). In addition, the patient benefited from apatinib chemotherapy, which was also consistent with sensitivity of the organoid. It is noted that Vascular inhibitors have also been shown to be effective against this type of tumor (25, 26).

Meanwhile, our research also indicated that combined MAA organoid based drug screening and high throughput sequencing could be a feasible way for personalized therapy. The combination of DNA sequencing with organoid high-throughput drug sensitivity tests has been widely used in individual chemotherapies of gastrointestinal tumors, such as colorectal and gastric cancers (27, 28). In our research, we found that the *TP53* gene underwent mutations, whereas the *APC, KRAS* genes did not undergo mutation, which is similar to other sequencing studies of MAA (10, 21). These results also implied gene mutations in MAA differ from those of colorectal cancer. The *FAT1* mutation is implicated in metastasis and drug resistance in various tumors, however, this mutation is sensitive to dasatinib (29). We found that dasatinib has a low IC_50_ value, implying that the organoid is sensitive to dasatinib. However, the efficacy of dasatinib in treatment of cancer of the appendix has never been reported before. Immunotherapies including check-points inhibitor are widely used for cancer therapy (30). However, its effectiveness against MAA has not been established. In this study, we found PDL1 staining on the organoid is negative, which imply the organoid may not be suitable for anti-programmed cell death-1 receptor (PD-1) therapy.

In addition, our research also provides a long-term culture MAA organoid line, which can be frozen cryopreserved for future research. As MAA cell lines shows slow growth rate and poor viability, it is necessary to develop new *in vitro* models to study the pathogenesis of MAA and for developing new treatment (22). The MAA organoid line retained the characteristics of the primary tumor. In addition, the MAA organoid had also been studied extensively in terms of pathological analysis, sequencing and drug screening, which could be a good alternative model of cell lines.

This study has several limitations. First, only one case was used, therefore, samples from different donors should be evaluated. Since HIPEC is an important treatment for MAA, a combination of organoids and PDX models may be explored to effectively select drugs for intraperitoneal therapy.

## Conclusions

We successfully established an MAA organoid, which exhibited the pathological characteristics and genetic mutations of the original tumor. Combined MAA organoid based drug screening and high throughput sequencing highlight the usefulness of MAA organoid models, as their matched patient response to chemotherapy and targeted therapy.

## Data Availability Statement

The datasets presented in this study can be found in online repositories. The names of the repository/repositories and accession number(s) can be found at: https://www.ncbi.nlm.nih.gov/ PRJNA808653.

## Ethics Statement

The studies involving human participants were reviewed and approved by the Seventh Affiliated Hospital of Sun Yat-sen University. The patients/participants provided their written informed consent to participate in this study. Written informed consent was obtained from the individual(s) for the publication of any potentially identifiable images or data included in this article.

## Author Contributions

YH, CZ, and WC conceived and designed the experiments. WH, JX, JW, MS, and XL contributed reagents, materials, and analysis tools. GL, XX, YX, SC, HL, and HW performed experiments and analysis and prepared the manuscript. All authors read and approved the final manuscript.

## Funding

This study was supported by Sanming Project of Medicine in Shenzhen (SZSM201911010), Shenzhen Key Medical Discipline Construction Fund (SZXK016), Shenzhen Sustainable Project (KCXFZ202002011010593), Guangdong Provincial Key Laboratory of Digestive Cancer Research (No. 2021B1212040006), and Shenzhen Fundamental Research Program (JCYJ20200109142605909). Research start-up fund of part-time PI, SAHSYSU (ZSQYJZPI202001).

## Conflict of Interest

The authors declare that the research was conducted in the absence of any commercial or financial relationships that could be construed as a potential conflict of interest.

## Publisher's Note

All claims expressed in this article are solely those of the authors and do not necessarily represent those of their affiliated organizations, or those of the publisher, the editors and the reviewers. Any product that may be evaluated in this article, or claim that may be made by its manufacturer, is not guaranteed or endorsed by the publisher.

## Supplementary Material

Supplementary Figure S1 **(A)** Overview of the design of this study. **(B)** Statistical analysis of immunohistochemical staining intensity of CDX-2, CK20, STAB2, PAX-8, and CK7 in organoid (passage 8) and tissue.

Supplementary Figure S2 Circos plots of the original tissue and cancer organoid.
