# Supplementary figures and images for: Organoids From Mucinous Appendiceal Adenocarcinomas as High-Fidelity Models for Individual Therapy
Source: Front Med (Lausanne). 2022 Jun 2;9:829033. doi: 10.3389/fmed.2022.829033 (PMC9201037; doi:10.3389/fmed.2022.829033)

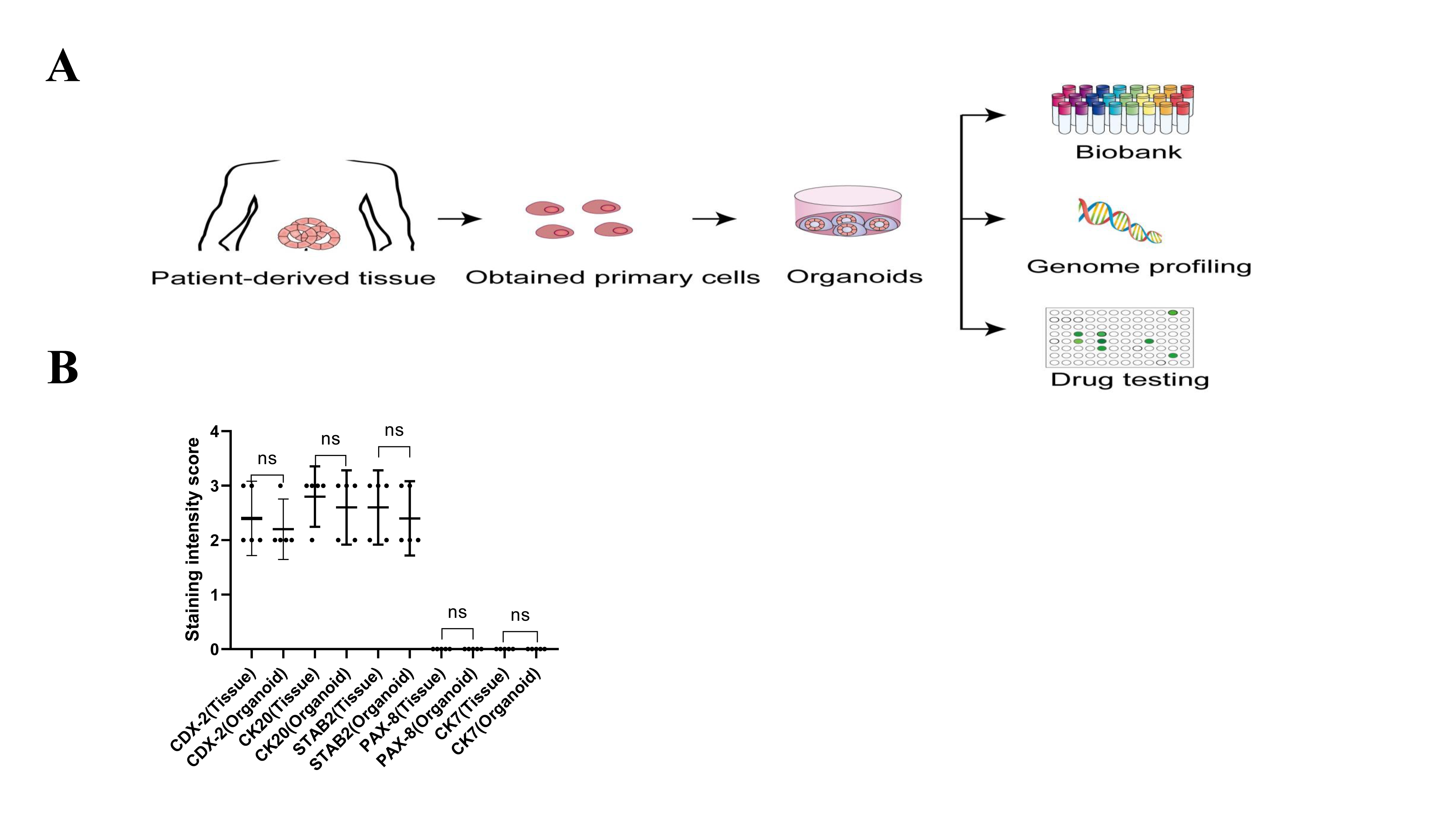

Supplement: Supplementary Figure S1 — (A) Overview of the design of this study. (B) Statistical analysis of immunohistochemical staining intensity of CDX-2, CK20, STAB2, PAX-8, and CK7 in organoid (passage 8) and tissue. [file Image_1.TIF]

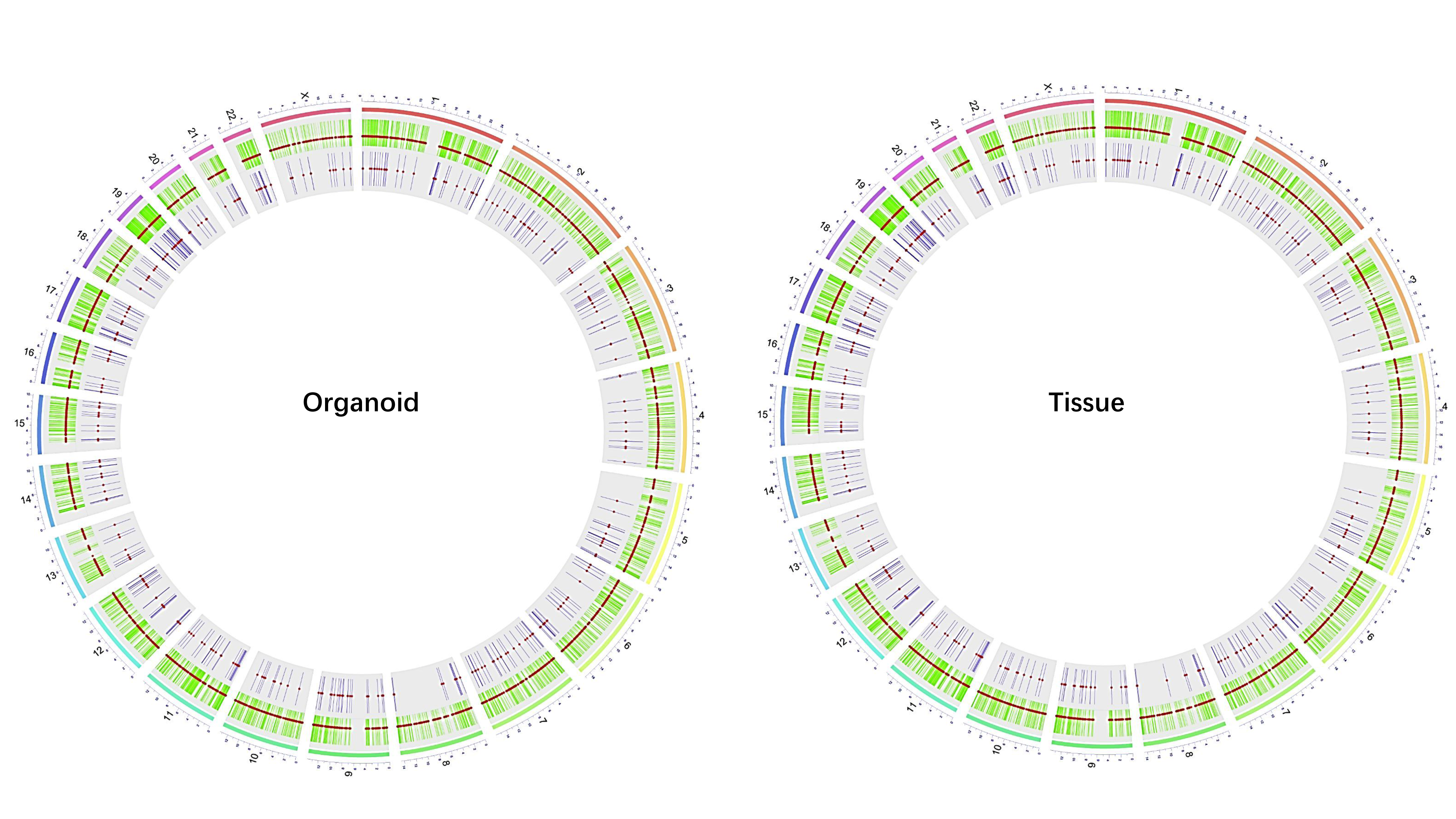

Supplement: Supplementary Figure S2 — Circos plots of the original tissue and cancer organoid. [file Image_2.TIF]
